# Passive surveillance of infectious bursal disease virus circulating in Egyptian chicken flocks in 2025

**DOI:** 10.1038/s41598-026-65194-0

**Published:** 2026-08-08

**Authors:** Amr H. Abd El-Fatah, Bouran Salama, Mariam O. Elnahhas, Noura M. Abo Shama, Mahmoud Shehata

**Affiliations:** 1 Vaccine Research and Development Department, EVA Pharma, 6th October City, Giza 12511 Egypt; 2 Center of Scientific Excellence for Influenza Viruses (CSEIV), National Research Centre, Cairo, 12622 Egypt

**Keywords:** IBDV, Very virulent, VP2 gene, Phylogenetics, Poultry disease, Egypt, Diseases, Immunology, Microbiology

## Abstract

Infectious bursal disease virus (IBDV) causes severe immunosuppression diseases in poultry, resulting in substantial economic losses for the global poultry industry. This study aimed to characterize circulating IBDV strains in Egyptian chicken flocks and assess their potential impact on poultry health and production. We conducted a passive surveillance study by collecting bursal samples from a total of 30 flocks, including commercial broilers, layers, and Baladi chickens, across nine Egyptian governorates in 2025. These flocks exhibited clinical signs of depression, along with kidney and bursal lesions indicative of IBDV infection. Pooled bursal homogenates were tested using RT-PCR with VP2-specific primers, revealing that 20 flocks (66.6%) tested positive for IBDV. Field and vaccine strains were distinguished by phylogenetic clustering of the VP2 hypervariable region (HVR) sequences against reference vaccine strains (D78, Winterfield 228, VAXXITEK), strains that cluster closely with reference vaccine strains are classified as vaccine-origin, whereas field strains are distinct branches indicate circulating wild-type or vvIBDV. Ten representative positive samples were inoculated in specific pathogen-free embryonated chicken eggs (SPF-ECE). The inoculated embryos exhibited haemorrhage, skull swelling, and liver necrosis with a pale-yellow appearance, along with congestion and thickening of the chorioallantoic membrane (CAM). Phylogenetic analysis of the partial VP2 gene classified the isolates according to the unified genogroup nomenclature: the majority of isolates belonged to the A3 (vvIBDV) genogroup, while one isolate clustered within the A2d (nVarIBDV) genogroup. These findings highlight the co-circulation of both virulent and variant IBDV strains in Egyptian chicken flocks, complicating disease control and demanding a continued surveillance and periodic re-evaluation of vaccination programs.

## Introduction

Infectious Bursal Disease Virus (IBDV) is globally distributed and highly contagious viral disease that induces severe immunosuppression in young chicks aged between 3 and 6 weeks, owing to its detrimental effect on lymphoid organs. The virus selectively destroys B-lymphocytes in the bursa of Fabricius, resulting in profound lymphoid depletion and progressive atrophy of the bursa of Fabricius. This immunosuppressive sequela renders affected flocks susceptible to secondary infections, resulting in significant economic losses for the global poultry industry^1^.

IBDV belongs to the genus *Avianbirnavirus* of the family *Birnaviridae.* It’s an icosahedral, non-enveloped and distinguished by its linear double-stranded bi-segmented RNA virus which is highly susceptible to rapid mutation rates due to the error-prone nature of its RNA-dependent RNA polymerase (RdRp), contributing to generation and circulation of new mutant or recombinant strains in chickens. A unified genotypic classification based on both genome segments A and B assigns strains to genogroups (A1–A6 for segment A and B1–B3 for segment B). Additionally, based on cross-neutralization, IBDV is divided into two serotypes: serotype 1 is pathogenic to chickens, while serotype 2 is non-pathogenic and infects turkeys. Pathotype classical IBDV (cIBDV), variant IBDV (varIBDV), very virulent IBDV (vvIBDV), attenuated (vaccine) strains, and the recently reported novel variant IBDV (nVarIBDV) are used to further distinguish strains within serotype 1 ^2^. The pathogenic vvIBDV strains predominantly belong to the A3 genogroup, whereas the Chinese/European novel variants cluster in the A2d genogroup. These pathotypes diverge considerably in their antigenic characteristics, degree of immunosuppression, and ability to escape vaccine-induced immunity^3,4^.

The bi-segmented double stranded RNA genome of IBDV is composed of segment A which is longer and contains two open reading frames (ORF) and encodes four viral proteins designated as VP2, VP3, VP4 and VP5, and segment B which only encodes the RNA-dependent RNA polymerase VP1. VP2, the main capsid protein of IBDV, is the vital protective immunogen and the sole viral protein capable of generating neutralizing antibodies, hence dictating the serotype and host immune response^5^. The antigenic factors responsible for diversity across IBDV strains are contained within VP2, particularly in its HVR. As this area is particularly prone to mutation, it drives antigenic drift, change in pathogenicity, vaccine escape and the creation of novel variations. Passive surveillance in which samples are collected from farms that voluntarily report clinical disease rather than through random, population-level sampling represents a pragmatic and cost-effective approach for detecting and characterizing circulating field strains in resource-limited settings. However, this is a passive surveillance study and is subject to sampling bias because only clinically affected flocks are represented; subclinical infections and healthy carriers are systematically under-sampled. Despite this limitation, passive surveillance provides timely molecular data that can guide vaccination policy and inform active surveillance priorities. Therefore, continuous molecular surveillance of VP2 is crucial to track such genetic changes, detect emerging field strains, and assess whether current vaccinations are effective against them^2,4^.

Infectious bursal disease outbreaks persist despite widespread vaccination in commercial broiler flocks because of the advent of highly virulent and variant IBDV strains that are genetically different from the vaccine strains currently in use. The Egyptian poultry industry suffers large financial losses because of these inconsistencies^6^. Thus, the purpose of this study is to identify, isolate, and genetically characterize circulating IBDV strains in Egyptian chicken flocks from different governorates to evaluate their possible effects on the health and productivity of poultry. The spread of both virulent and antigenically different mutant strains challenges disease control efforts and casts doubt on the efficacy of current immunization regimens. These results emphasize the vital necessity of continuous molecular surveillance and frequent assessment of vaccination formulations to provide sufficient defense against evolving field strains^7^.

## Materials and methods

### Sampling

**Fig. 1:**
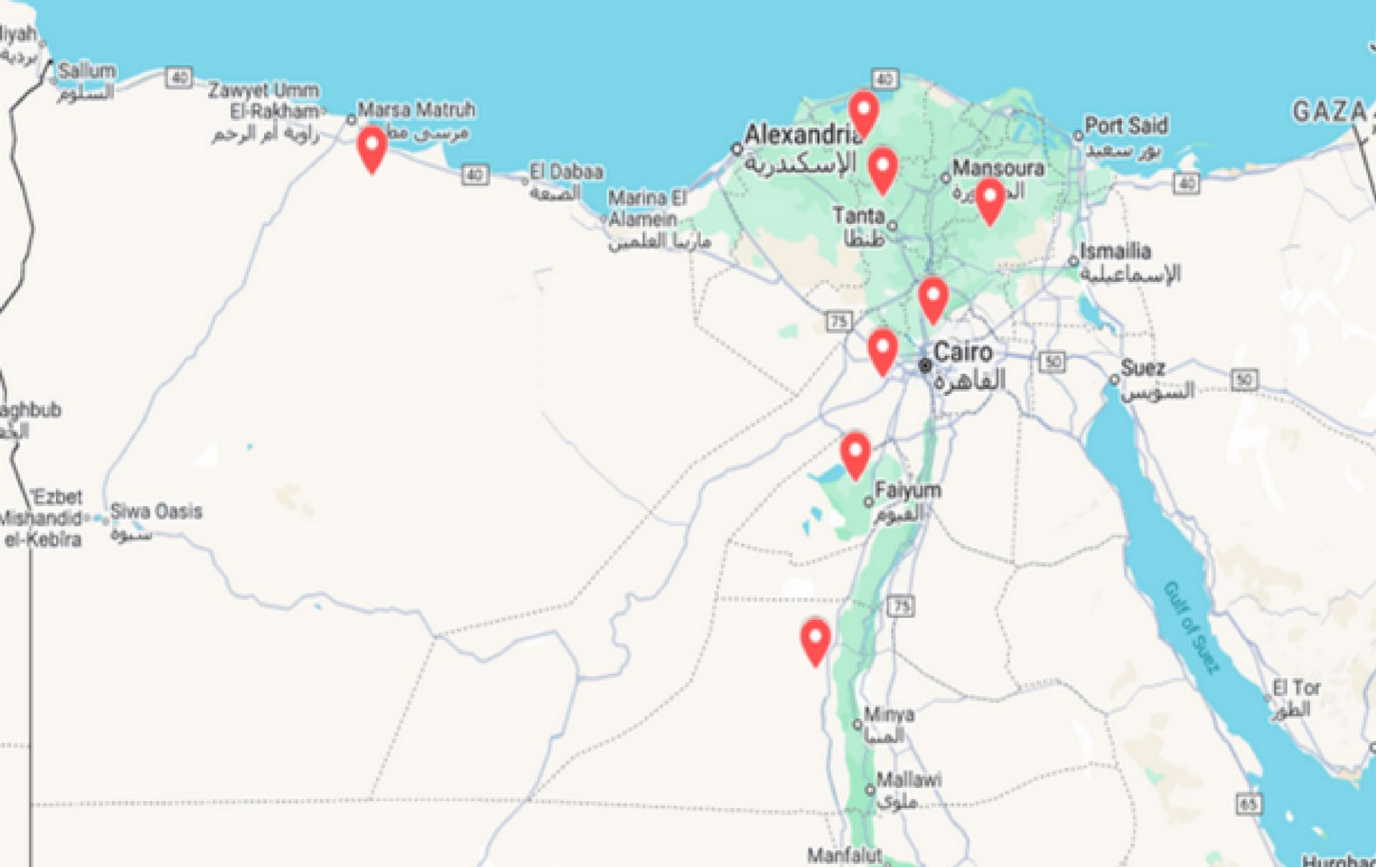
Egypt map shows the sampling sites in our study (Giza, Kalyoubia, Kafr Elsheikh, Marsa Matrouh, Sharkia, Gharbya and El-Menia).

Bursal tissue was harvested from diseased and freshly deceased chicks across 20 poultry farms located in nine Egyptian governorates (El-Qalubiya, Alexandria, Kafr-Elshiekh, El-Gharbia, El-Sharqia, Giza, El-Menya, Marsa Matrouh, and El-Fayoum), as shown in Fig. 1. Because several farms maintained more than one independent production unit (flock), a total of 30 flocks were ultimately examined. Sample selection criteria were applied as follows: only flocks aged 3–6 weeks presenting with clinical signs consistent with IBD (acute depression, nephritis, and bursal enlargement) and/or elevated mortality were included; flocks without clinical signs were excluded. Samples were collected from clinically affected or freshly dead birds submitted by farm owners for routine diagnostic investigation; no experimental infection was performed. Birds were handled according to institutional animal welfare guidelines, and those requiring euthanasia were humanely euthanized by cervical dislocation performed by trained personnel.

For analysis, specimens were divided: one portion was fixed in buffered formalin for histopathological examination, while the other was frozen for isolation. To isolate IBDV, the bursae were homogenized using sterile sand and phosphate-buffered saline (PBS) supplemented with penicillin and streptomycin (1000 µl/ml). The homogenates were centrifuged at 3000xg for 10 min, and the resulting supernatants were passed through a filter and stored at − 80 °C until further processing^8^.

Identification of IBDV using reverse transcription-polymerase chain reaction (RT-PCR).

Viral RNA was purified from bursal tissues using the Gene JET Viral DNA/RNA Purification Kit (Thermo Scientific), followed by cDNA synthesis using the Maxima H Minus Double-Stranded cDNA Synthesis Kit (Thermo Scientific). In-house primers (Forward: 5′-TCACCGTCCTCAGCTTACCCACATC-3′; Reverse: 5′-ATTTGGGATCAGCTCGAAGTTGC-3′) were used to amplify a 639 bp fragment covering the VP2 at HVR via conventional RT-PCR on a StepOnePlus™ thermal cycler (Applied Biosystems). A previously confirmed IBDV-positive field sample served as the positive control in every run; a no-template (nuclease-free water) reaction was included as the negative control. The thermal protocol included 35 cycles of 94 °C (10 s), 64 °C (30 s), and 72 °C (50 s), preceded by a 5-min activation at 95 °C and a final 10-min extension at 72 °C. PCR products were visualized on a 1.5% ethidium bromide-stained agarose gel (0.5 µg/ml) at 120 V for 30 min, alongside a GeneRuler 100 bp Plus DNA Ladder (Thermo Scientific™). It is acknowledged that the 639 bp VP2-HVR fragment is sufficient for genogroup classification and phylogenetic analysis but is insufficient for definitive recombination detection or complete vaccine-strain discrimination would require longer amplicons or complete-segment sequencing^4^.

### Virus isolation

Bursal homogenates (0.2 ml) were inoculated onto the CAM of 11–13-day-old SPF-ECE (Koum-Oshiem farm, Egypt) at passage 1. After an incubation period of 3–5 days at 37 °C, the eggs were cooled at 4 °C overnight. CAMs were harvested, washed three times in sterile PBS, collected in 50 ml sterile tubes, and subjected to sonication in a water bath. positive samples were passaged 2–4 times on CAM until consistent lesions (hemorrhage, edema) appeared^4^. Then, sequence analysis was done using viral harvest from passage 3 on SPF eggs.

### Sequencing and sequence analysis of VP2 of IBDV

RNA extracted from CAM of ten IBDV isolates was subjected to RT-PCR using the previously described primers and cycling conditions. The resulting PCR amplicons of the expected size were excised from the agarose gel and purified using the QIAquick Gel Extraction Kit (Qiagen, Hilden, Germany). Sequencing was performed utilizing the ABI PRISM^®^ Big Dye™ Terminators v3.1 Cycle Sequencing Kit and an ABI PRISM^®^ 3130 genetic analyzer equipped with 80 cm capillaries (Applied Biosystems, Foster City, CA, USA). Sequence editing was conducted with SeqScape Software v2.5 (Applied Biosystems), while consensus sequence assembly and alignment trimming were carried out using the Lasergene DNASTAR suite (DNASTAR Inc., Madison, WI, USA) via the Clustal W method. Following the protocol outlined by^9^, the DNAstar-MegAlign program was used to generate pairwise sequence alignments and calculate nucleotide identity divergence matrices. Phylogenetic trees were constructed using MEGA 11.0 employing the Neighbor-Joining (NJ) method with the Maximum Composite Likelihood substitution model; model selection was performed using the built-in model test feature within MEGA 11, and 1000 bootstrap replicates were used to assess node support. Reference sequences, including Egyptian IBD viruses and other international strains, were retrieved from the National Centre for Biotechnology Information (NCBI).

To assess the potential functional impact of the observed amino acid substitutions, we mapped residues 220, 270, and 299 onto a three-dimensional model of the VP2 capsid protein (generated using Swiss-Model with template PDB ID 6P6X and visualized with PyMOL software).

## Results

### Farm history and mortality rates of IBDV-affected chicken flocks

Chickens on the studied farms with clinical signs that included swollen and hemorrhagic bursae, as well as hemorrhaging in the thigh and breast muscles. As shown in Table 1, mortality was assessed over a seven-day period following the initial increase in deaths, with rates fluctuating between 3% and 20%.

While the observational nature of this passive surveillance study precluded a formal statistical correlation analysis, a descriptive summary of the data highlights distinct trends regarding vaccine breakthrough (Table 1). Broiler flocks vaccinated with the recombinant vector vaccine (Vaxxitek) generally presented with moderate mortality rates ranging from 3% to 9% and lesion scores of mild (+) to moderate (++). In contrast, flocks receiving live ‘Hot’ or Intermediate Plus vaccines (e.g., Bursin Plus, Winterfield 228) exhibited the widest range of mortality, peaking at 20% (flock EV7) and frequently showing severe lesion scores (+++).

Notably, the sole flock infected with the variant A2d strain (EV10) had received a protocol including an inactivated vaccine and an IBDL live booster, yet still experienced high mortality (12%) and severe lesions (+++). This summary indicates that while clinical disease occurred in flocks vaccinated with all major vaccine types available in Egypt, the most severe pathological outcomes were associated with the antigenically distinct A2d variant and flocks utilizing ‘Hot’ vaccine strategies, suggesting a variable degree of cross-protection. Furthermore, vaccination protocols varied considerably across the flocks, utilizing various products including Transmmune, Innovax, Bursaplex, and Intermediate strains administered at different ages.

**Table 1:** Represent data of farms in our study.

Flock code	Chicken type	Governate	Age (days)	Flock size	vaccination		Mortality (%)	Severity of lesions
					Age	Vaccine		
EV1	Broiler	Alexandria	24	5,000	1,10	Transmmune, Intermediate	8	++
EV2	Broiler	Giza	24	20,000	1	VAXXITEK	6	+
EV3	Broiler	Beheira	30	110,000	1, 8	VAXXITEK, D78	3	++
EV4	Layers	Al-Qalyubia	22	8,000	13	Winterfield 228	6	++
EV5	Layers	Al-Qalyubia	39	4,600	14	IBDL (Ceva)	6	+++
EV6	Broiler	Giza	31	149,500	1, 13	VAXXITEK, Intermediate	3	++
EV7	Broiler	Al-Qalyubia	30	5,000	14	Bursin Plus	20	++
EV8	Broiler	Al-Qalyubia	34	10,500	1, 15	Innovax, D78	3	++
EV9	Broiler	Al-Qalyubia	24	11,000	1, 12	Bursaplex, Intermediate	2	+
EV10	Layers	Al-Qalyubia	42	4,500	7, 12	Inactivated, IBDL (Ceva)	12	+++
EV11	Layers	Al-Qalyubia	30	24,000	8	Inactivated	8	+++
EV12	Broiler	Alexandria	30	8,000	12	Intermediate	8	++
EV13	Broiler	Al-Qalyubia	32	7,000	1	Transmmune	6	++
EV14	Baladi	Al-Mansoura	36	10,000	12	Intermediate Plus	7	+
EV15	Broiler	Al-Mansoura	28	9,000	1, 8	VAXXITEK, Intermediate	4	++
EV16	Baladi	Al-Gharbia	30	10,000	12	Intermediate Plus	6	++
EV17	Baladi	Al-Mansoura	25	7,000	12	Intermediate Plus	5	++
EV18	Broiler	Al-Qalyubia	25	3,500	12	Hot	8	++
EV19	Broiler	Giza	24	20,000	1	VAXXITEK	5	+
EV20	Broiler	Giza	24	20,000	1	VAXXITEK	9	+
EV21	Broiler	El-Sharqia	28	15,000	1, 14	Transmmune, D78	5	++
EV22	Broiler	Kafr-Elshiekh	32	12,000	12	Intermediate	7	++
EV23	Layers	Giza	40	5,000	10, 20	Winterfield, Inactivated	4	+
EV24	Broiler	Beheira	26	25,000	1, 15	VAXXITEK, Intermediate	9	++
EV25	Baladi	El-Dakahlia	35	8,000	12	Intermediate Plus	6	++
EV26	Broiler	Alexandria	30	18,000	1, 10	Bursaplex, Hot	12	+++
EV27	Broiler	El-Menya	22	10,000	1	Transmmune	3	+
EV28	Layers	El-Gharbia	45	4,000	14	IBDL (Ceva)	5	++
EV29	Broiler	El-Fayoum	29	22,000	1, 16	VAXXITEK, D78	6	++
EV30	Baladi	Marsa Matrouh	33	6,000	14	Intermediate	8	++

### Molecular screening of IBDV using RT-PCR

A positivity rate of 66.6% (20 out of 30 flocks) was observed when all flocks across the 20 farms were screened for IBDV using VP2-specific RT-PCR.

**Fig. 2:**
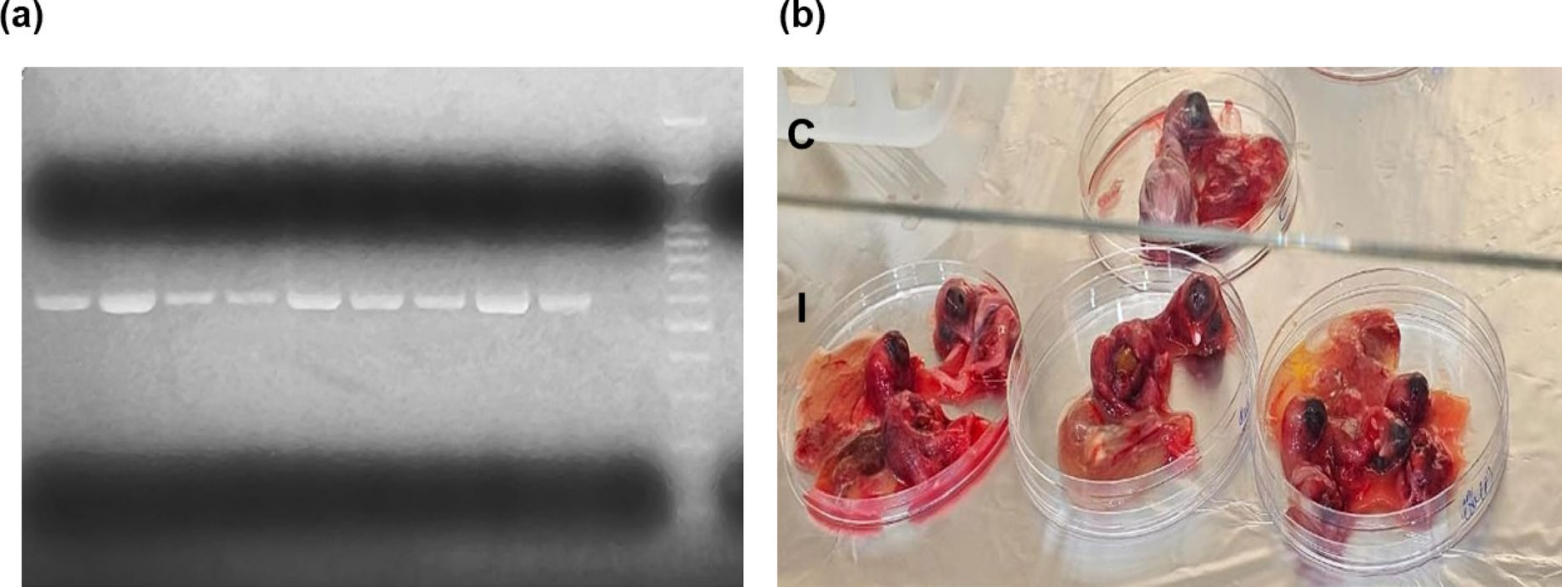
(**a**) Gel electrophoresis of the VP2 gene fragments amplified by RT-PCR. The bands correspond to the expected size of 639 bp. Lane 1 to 9 IBDV strains were isolated in current study, while lane 10 is a negative control. (**b**) Upon harvesting, the embryos showed signs of hemorrhage and skull swelling, and the CAMs were found to be thickened and congested. Figure panels are as follows: (I) infected with severe symptoms, (C) control.

### IBDV isolation

Ten samples (EV1, EV2, EV3, EV4, EV5, EV6, EV7, EV8, EV9, and EV10) that previously tested positive via RT-PCR were inoculated onto the CAM of 11-day-old specific SPF-ECE. Compared to negative control, the inoculated embryos exhibited lesions indicative of IBDV replication, including hemorrhage, skull swelling, and pale-yellow liver necrosis. Additionally, the harvested CAMs appeared thick and congested (Fig. 2b). Liver and CAM tissues were collected from the embryos and subjected to VP2 amplification using RT-PCR. This successfully yielded 639 bp bands (Fig. 2a), which were subsequently purified and sent for sequencing.

### Sequence analysis of the hyper variable region of VP2

Isolates with accessions (PX789557–PX789566) revealed specific patterns of amino acid (AA) substitution within the vvIBDV A3 lineage, particularly within hydrophilic peaks and structural domains known to affect capsid antigenicity. In the Major Hydrophilic Peak A region, the majority of isolates exhibited high conservation ≥ 95% sequence identity across isolates, all 2025 isolates possessed phenylalanine (F) at residue 220, whereas the Egyptian 2019 reference strain contained tyrosine (Y), indicating a substitution relative to the earlier reference strain. Isolates displayed a unique 220 F substitution; this conservative change suggests a minimal evolutionary divergence, marking this isolate as a relatively ancestral genotype within the Egyptian vvIBDV population. Furthermore, isolate EV9/2025 with accession PX789565 was characterized by a unique substitution position 270E, representing a distinct evolutionary branch. Isolate EV4/2025 (PX789560) has a substitution at 299 N in minor hydrophilic peak 3, and isolate EV6/2025 (PX789562) exhibited changes in major hydrophilic peak A 221 K, 222T, also at Major hydrophilic peak B 318D. Isolate EV10/2025 (PX789566) proved to be the most divergent of the 2025 population, uniquely carrying non-conservative charged AA changes also a complex substitution pattern. These complex mutation profiles, ranging from the relatively minor changes to the significant divergence ≥ 5% difference from reference or vaccine strains, illustrate the ongoing genetic evolution and accumulation of independent mutations within the local IBDV population.

**Fig. 3:**
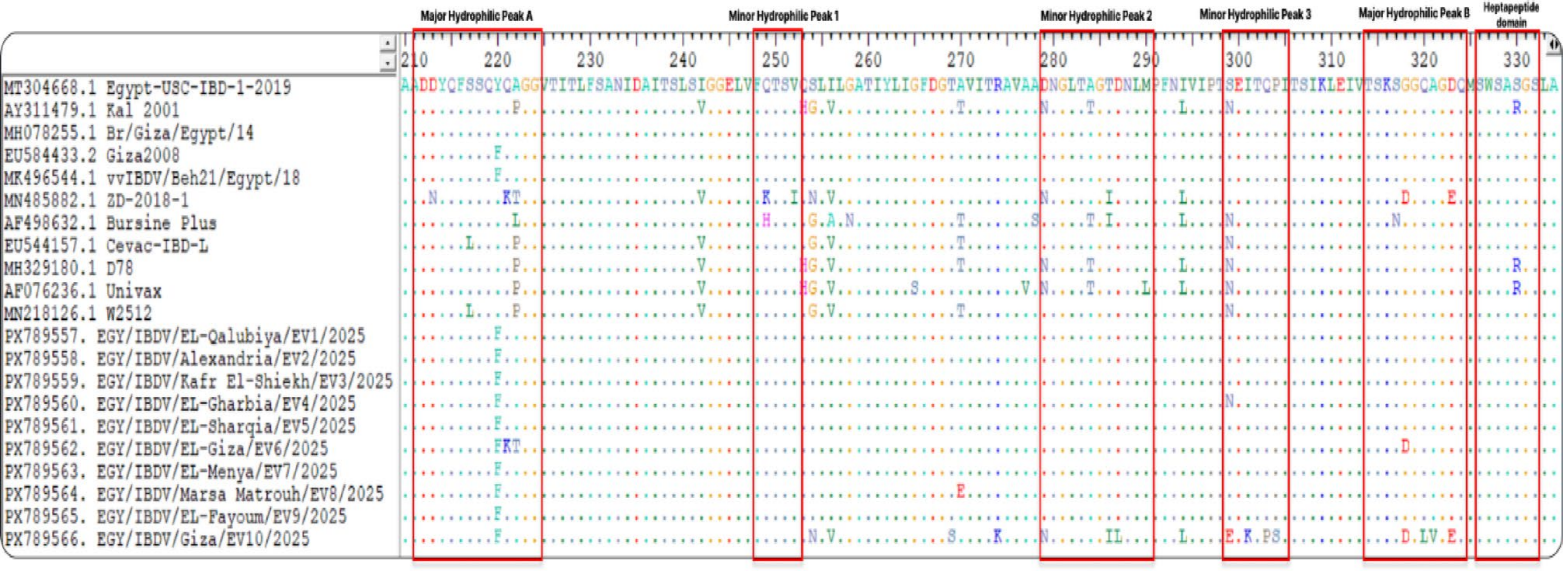
Deduced amino acid alignment of the VP2 hypervariable region (spanning residues 211 to 350) of the analyzed IBDV strains. Dots indicate amino acid positions that are conserved relative to the reference sequence. Red boxes demarcate the major and minor hydrophilic peaks.

The shaded positions (221, 222, 254, 286, 318, 321, and 323) lie within or adjacent to the major and minor hydrophilic peaks of VP2, which are antigenic epitopes known to influence neutralizing antibody binding and immune escape. Among the 2025 isolates, EV10 exhibits the most divergence, clustering within genogroup A2d (nVarIBDV), while EV6 shows distinct substitutions (221 K, 222T, and 318D) within the otherwise homogenous A3 vvIBDV group. Importantly, the vaccine strains D78 and Winterfield 228 differ substantially from these Egyptian field isolates at multiple positions (220 S/F, 221 K/Q, 222P/A/T), highlighting an antigenic gap that may contribute to vaccine breakthroughs under field conditions. The analysis of AA positions 220, 221, 222, 254, 256, 279, 286, 318, 321, and 323 revealed a high level of conservation across the Egyptian variants (EV1–EV10), with limited substitutions observed as illustrated in Fig. 3; Table 2. At position 220, phenylalanine (F) was detected in all 2025 Egyptian variants (EV1–EV10). This differs from the Egypt 2019 reference strain, which carries tyrosine (Y) at this position; the F substitution relative to the 2019 reference may therefore represent a locally emerging mutation associated with immune escape in the circulating 2025 population. Position 221, also linked to immune escape, showed glutamine (Q) conservation in all variants except EV6, which exhibited a lysine (K) substitution. Position 222, involved in immune escape as well as viral replication and virulence, was predominantly alanine (A), with a threonine (T) substitution detected only in EV6, while the Egypt 2019 reference retained alanine. At position 254, serine (S), associated with immune escape, was conserved across EV1–EV9, whereas EV10 showed an asparagine (N) substitution; the reference sequence remained serine. Position 256, related to viral replication and virulence, showed isoleucine (I) conservation in EV1–EV9, with a valine (V) substitution in EV10, consistent with the reference isoleucine. At position 279, linked to attenuation and surface charge, aspartic acid (D) was conserved in EV1–EV9, while EV10 displayed an asparagine (N) substitution; the Egypt 2019 reference retained aspartic acid. Position 286, associated with immune escape, showed threonine (T) conservation across EV1–EV9, with an isoleucine (I) substitution in EV10, matching the reference threonine. At position 318, glycine (G) was conserved in most variants, except EV6 and EV10, which exhibited an aspartic acid (D) substitution; the reference sequence remained glycine. Position 321, involved in immune escape and loop charge, was conserved as alanine (A) across EV1–EV9, with a valine (V) substitution in EV10, while the reference also showed alanine. Finally, at position 323, associated with virus assembly and immune escape, aspartic acid (D) was conserved across EV1–EV9, whereas EV10 exhibited a glutamic acid (E) substitution, consistent with the reference aspartic acid.

**Fig. 4:**
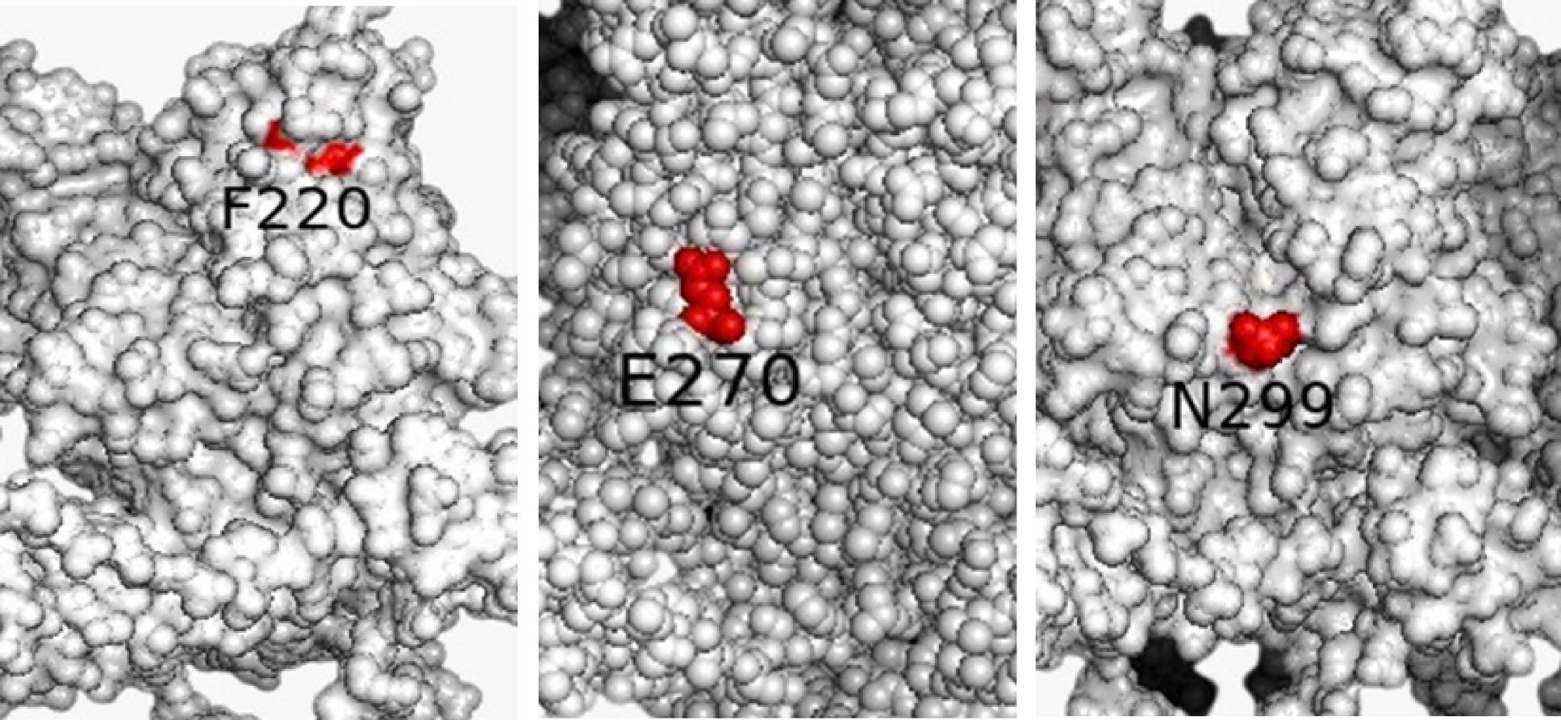
Shows 220 F mutation located in the P_BC_ loop (antigen-antibody affinity affecting region), 270E in domain I and 299 N lies within a loop that contacts the antigen‑binding region of neutralizing antibodies.

As shown in Fig. 4, residue 220 F is located within the P_BC_ loop (antigen-antibody affinity affecting region), which forms part of a major neutralizing epitope on the outer surface of the virion^10^. Residue 270E lies within domain I of the VP2 hypervariable region, a structural element that contributes to the conformational integrity of the capsid projection domain. Finally, residue 299 N is situated near the D78 vaccine strain binding interface, based on structural alignment of the 2025 VP2 with the D78 VP2 crystal structure.

**Table 2:** Amino acid residues at key positions within the VP2 hypervariable region (HVR) of 2025 Egyptian IBDV isolates (EV1–EV10) compared to an Egyptian reference strain (Egypt 2019) and two representative vaccine strains (D78 and Winterfield 228).

Isolate / Strain	220	221	222	254	256	279	286	318	321	323
EV1	F	Q	A	S	I	D	T	G	A	D
EV2	F	Q	A	S	I	D	T	G	A	D
EV3	F	Q	A	S	I	D	T	G	A	D
EV4	F	Q	A	S	I	D	T	G	A	D
EV5	F	Q	A	S	I	D	T	G	A	D
EV6	F	**K**	**T**	S	I	D	T	**D**	A	D
EV7	F	Q	A	S	I	D	T	G	A	D
EV8	F	Q	A	S	I	D	T	G	A	D
EV9	F	Q	A	S	I	D	T	G	A	D
EV10	F	Q	A	**N**	**V**	**N**	**I**	**D**	**V**	**E**
Egypt 2019 (reference)	**Y**	Q	A	S	I	D	T	G	A	D
Vaccine: D78 (classical)	**S**	**K**	**P**	**G**	**V**	**N**	**I**	**S**	**P**	**D**
Vaccine: Winterfield 228 (intermediate)	**S**	**K**	**P**	**S**	**V**	**N**	**I**	**G**	**A**	**E**

Bold letters; key mutantions versus reference and vaccine strains.

**Fig. 5:**
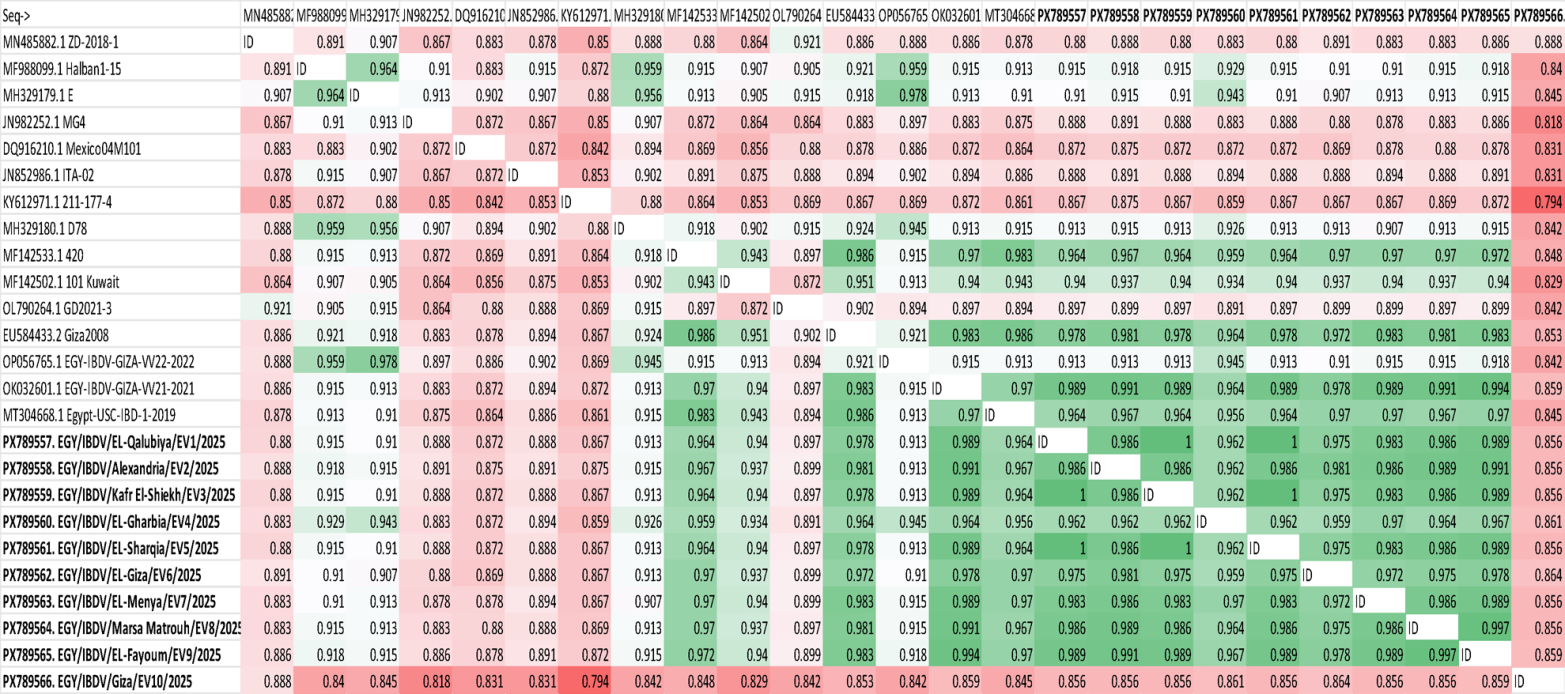
Identity matrix between IBDV isolates and other representative reference strains.

The identity matrix indicates a high degree of genetic similarity among the recent Egyptian isolates (Fig. 5). The diagonal values represent 100% self-identity. The cells shaded in green (representing values > 0.90 or > 0.95) likely show that the field isolates share significant sequence identity with one another. This suggests a clonal expansion of a specific viral population across the different governorates, indicating that a dominant strain is circulating within the Egyptian poultry population during 2025. The identity matrix analysis quantitatively confirms that the 2025 Egyptian IBDV isolates are highly homogenous and closely related to very virulent strains, while remaining genetically distinct from classical vaccine types. The 2025 vvIBDV isolates showed 91.2–93.8% nucleotide identity with D78 vaccine strain, while the variant isolate EV10/2025 showed only 89.5% identity, indicating significant antigenic divergence as shown in Fig. 5.

### Phylogenetic analyses for hyper variable region of VP2

**Fig. 6:**
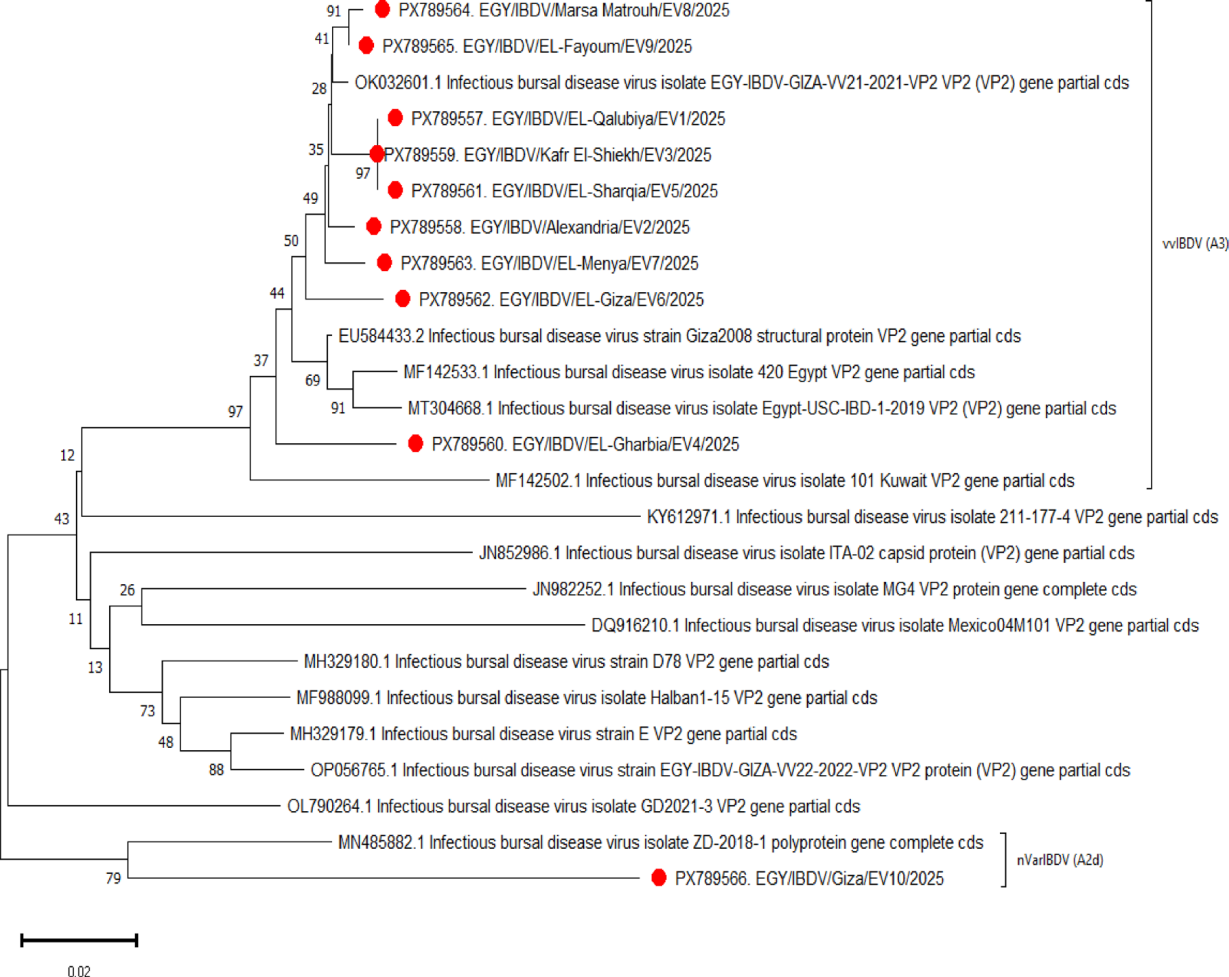
The phylogenetic tree was constructed from VP2 nucleotide sequences. The nucleotide sequences analyzed using MEGA 11.0 with neighbor-joining phylogenetic trees method using the Maximum Composite Likelihood model with 1000 bootstrap replicates. The sequenced samples in our study were in red bullets.

The phylogenetic analysis of the HVR sequences generated nine major clusters. The tree was constructed using the Neighbor-Joining method with the Maximum Composite Likelihood model and 1000 bootstrap replicates in MEGA 11. Most isolates clustered within the very virulent A3 genogroup, sharing a clade with reference strains Egypt-USC-IBD-1-2019. This group included isolates with accession numbers from PX789557 to PX789565. However, the isolate Egy/IBDV/Giza/EV10/2025 accession PX789566 was an outlier; it did not associate with the vvIBDV strains but instead clustered with Chinese variant strains in the A2d genogroup (Fig. 6).

## Discussion

The present study provides a comprehensive update on the epidemiological, and molecular status of IBDV circulating in Egyptian chicken flocks in 2025. Through passive surveillance across nine governorates, we demonstrated a high prevalence of IBDV (66.6%) in flocks exhibiting clinical signs of immunosuppression and mortality, despite the implementation of diverse vaccination protocols. The isolation of IBDV in embryonated chicken eggs and subsequent phylogenetic analysis of the VP2-HVR revealed the co-circulation of very virulent IBDV (vvIBDV) and distinct variant strains, highlighting the complex and evolving nature of the virus in the region.

The high detection rate (66.6%) of IBDV in flocks showing clinical signs aligns with earlier reports from Egypt and the broader Middle East, where IBD remains endemic despite widespread vaccination^11,12^. The variation in mortality (2–20%) and lesion severity across flocks likely reflects differences in bird age, breed, immune status, and critically the type and timing of vaccination. The diverse vaccination protocols documented here, ranging from live intermediate to recombinant and inactivated vaccines, highlight the industry’s attempt to adapt to field pressure. However, the continued outbreaks suggest that vaccine protection is often incomplete, possibly due to antigenic mismatch between vaccine strains and circulating field viruses^13^.

The clinical presentation and pathological findings observed in this study, including swollen and hemorrhagic bursae, nephritis, and high mortality rates up to 20%, align with the characteristic pathogenicity of vvIBDV. Notably, the most severe lesions and highest mortality were recorded in flocks vaccinated with commercially available live and inactivated vaccines, Bursin Plus, VAXXITEK, and IBDL. This observation raises concern about potential reduced vaccine efficacy under field conditions. Clinical disease occurred despite vaccination, and that reduced vaccine efficacy is a possible explanation. However, it must be emphasized that, in the absence of controlled challenge experiments, vaccine failure cannot be confirmed solely from passive surveillance data; concurrent factors such as maternal antibody interference, cold-chain breaches, or improper vaccine administration cannot be excluded^14^. The farm history data indicates that while vaccination is widely practiced, the efficacy of current immunization regimens is inconsistent. This failure is likely not due to improper administration alone but is fundamentally rooted in the genetic and antigenic divergence between the field strains and the vaccine strains used.

As shown in Fig. 4, substitution at residue 220 F which located at P_BC_ loop can directly alter antibody binding affinity, and the change from tyrosine (Y) in the 2019 reference to phenylalanine (F) in all 2025 isolates represents a conservative but potentially significant modification of the epitope’s hydrophobic character^12,15^. The introduction of a negatively charged glutamic acid (E) at this position 270 may alter local electrostatic surface properties, potentially reducing recognition by vaccine induced antibodies that were raised against strains carrying a neutral or opposite charge at this site^16^. The asparagine (N) at position 299 replaces the residue present in most vvIBDV isolates and lies within a loop that contacts the antigen-binding region with neutralizing antibodies. Changes at this proximal site may sterically hinder or destabilize antibody binding^4^. Collectively, the spatial distribution of these three mutations affecting the P_BC_ loop, domain I, and the D78 interaction surface suggests that they could act synergistically to facilitate immune escape from commonly used vaccines^17^.

Phylogenetic analysis of the VP2-HVR revealed that most Egyptian isolates from 2025 clustered within the vvIBDV genogroup A3, closely related to earlier Egyptian strains such as Egypt-USC-IBD-1-2019. This indicates the persistent circulation and possible local evolution of vvIBDV in the region. Notably, one isolate Egy/IBDV/Giza/EV10/2025 clustered separately within the A2d genogroup, which includes Chinese variant strains. This finding raises the possibility that an antigenically distinct variant has been introduced into Egypt, potentially via international trade of live poultry, hatching eggs, or contaminated equipment. While this hypothesis is consistent with existing literature documenting the spread of A2d-type variants across the Middle East and North Africa including the first report of novel variant IBDV (A2dB1b) in Egypt by Legnardi et al. ^7^. Epidemiological confirmation would require whole-genome sequencing and trace-back investigations. The vaccines currently used in Egypt span several categories: live attenuated classical strains (D78, Intermediate, Intermediate Plus), immune complex/vector vaccines (VAXXITEK HVT + IBD, Bursaplex), inactivated oil-emulsion vaccines, and live strains (Winterfield 228, Bursin Plus). Of these, VAXXITEK and D78 are based on classical serotype 1 strains with VP2 sequences substantially divergent from both vvIBDV (A3) and A2d-genogroup variants^18^. The amino acid differences documented in the present study at positions 221, 222, and 318 fall within established neutralizing epitope regions, supporting the possibility that reduced cross-neutralization contributes to the observed vaccine breakthroughs^19^. An in vivo challenge and cross-neutralization studies are recommended to formally test this hypothesis.

The AA substitutions observed within the major and minor hydrophilic peaks of VP2 are of particular concern. These regions are critical for virus neutralization and host immune recognition^20^. The unique substitutions at positions 220 F, 270E, 299 N, and 318D among our isolates may represent adaptive mutations that influence viral antigenicity and potential vaccine escape. It’s crucial to remember that antigenic drift in this context refers to slow, mutation-driven changes in surface-exposed epitopes; neutralization assays using antisera raised against the reference and divergent strains are necessary to definitively demonstrate antigenic drift, but they were outside the purview of this surveillance study. For instance, changes at positions 221Q and 222 A have been previously associated with altered binding of neutralizing antibodies^10,21^. The divergence of isolate Egy/IBDV/Giza/EV10/2025, with its multiple non-conservative substitutions, underscores the capacity of IBDV for rapid evolution and emergence of novel variants in the field. The high level of AA conservation observed within the VP2-HVR among the Egyptian variants suggests strong structural and functional constraints on this major antigenic determinant. Most residues previously associated with immune escape, virulence, and viral replication remained unchanged relative to the Egypt 2019 reference strain, indicating limited antigenic drift in the circulating strains. The few substitutions detected particularly at positions 221, 222, and 318, which are located within or adjacent to known neutralizing epitopes may reflect localized adaptive changes with potential effects on antibody recognition. Variations observed in the A2d variant EV10/2025 lacks the classical and vvIBDV strains VP2 markers but carries Q253H (domain I), A284T, and V321I (domain II). These residues lie within neutralizing epitopes; their substitutions likely alter conformational epitopes recognized by vaccine-induced antibodies such as D78 or VAXXITEX vaccine, explaining reduced cross-protection^16,21^. EV10 at multiple VP2-HVR positions could indicate ongoing viral microevolution; however, the predominantly conservative nature of these substitutions suggests preservation of capsid stability and viral fitness. Collectively, these findings support the continued relevance of current VP2-based vaccine strategies while highlighting the importance of molecular surveillance to monitor emerging mutations that may influence immune escape or pathogenicity^4^.

The high genetic homogeneity (> 90% identity) among most vvIBDV isolates points to a clonal expansion of a dominant strain across multiple governorates. This pattern is consistent with the highly contagious nature of IBDV and the movement of birds, equipment, or personnel within the poultry sector. In contrast, the lower identity scores (~ 80%) between field isolates and classical vaccine strains D78 highlight a significant antigenic gap that may explain vaccination failures in the field. Similar findings have been reported in studies from Asia and Latin America, where vvIBDV and variant strains have shown reduced cross-protection with traditional vaccines^22^.

The isolation of IBDV in SPF embryonated eggs and the associated embryonic lesions (hemorrhage, liver necrosis, CAM thickening) confirm the virulence of the circulating strains. These pathological features are consistent with those described for vvIBDV and some variant strains, further supporting their pathogenic potential^23^.

## Conclusion

Our study demonstrates the co-circulation of genetically distinct IBDV strains in Egypt, including vvIBDV and a Chinese-like variant. The observed mutations in the immunodominant VP2 region may contribute to antigenic drift and reduced vaccine efficacy. These results reinforce the need for continuous molecular surveillance to monitor the emergence and spread of novel strains. Additionally, vaccination strategies in Egypt should be periodically re-evaluated, considering the inclusion of antigenically matched or multivalent vaccines to broaden protection. Future studies should include in vivo challenge experiments to assess the pathogenicity of these isolates and the cross-protective efficacy of current vaccines.

## Data Availability

All data generated or analyzed during this study are included in this published article. Data can be requested from corresponding author by email. Sequences were available through NCBI database with the following accessions (PX789557–PX789566).

## Abbreviations

AA: Amino acid
CAM: ChorioAllantoic membrane
cIBDV: Classical IBDV
ECE: Embryonated chicken eggs
HVR: Hypervariable region
IBDV: Infectious bursal disease virus
NCBI: National Centre for Biotechnology Information
nVarIBDV: Novel variant IBDV
ORF: Open reading frames
PBS: Phosphate-buffered saline
RdRp: RNA-dependent RNA polymerase
SPF: Specific pathogen-free
varIBDV: Variant IBDV
vvIBDV: Very virulent IBDV
